# Evaluation of instrumental variable method using Cox proportional hazard model in epidemiological studies

**DOI:** 10.1016/j.mex.2023.102211

**Published:** 2023-05-11

**Authors:** Md. Jamal Uddin, Tanvir Ahammed, A.Z.M. Hasan Kabir

**Affiliations:** a Department of Statistics, Shahjalal University of Science and Technology, Sylhet 3114, Bangladesh; b Department of General Educational Development (GED), Daffodil International University, Dhaka, Bangladesh; c Department of Mathematics, Comilla Victoria Government College, Cumilla, Bangladesh

**Keywords:** Instrumental variable analysis, Cox proportional hazard model, Survival data, Confounder, Causal Research, Bias, Simulation studies

## Abstract

The instrumental variable (IV) method with a Cox proportional hazard (PH) model has been used to evaluate treatment effects in epidemiological studies involving survival data. The effectiveness of the IV methods in these circumstances has yet to be fully understood, though. The study aimed to evaluate the performance of IV methods using a Cox model. We evaluated the validity of treatment effects estimated by two-stage IV models using simulated scenarios with varying confounder strengths and baseline hazards. Our simulation demonstrated that when observed confounders were not taken into account in the IV models, and the confounder strength was moderate, the treatment effects based on the two-stage IV models were similar to the true value. However, the effect estimates diverged from the true value when observed confounders were taken into account in the IV models. In the case of a null treatment effect (i.e., hazard ratio=1), the estimates from the unadjusted and adjusted IV models (only two-stage) were close to the true value. The implication of our study findings is that the treatment effects obtained through IV analyses using the Cox PH model remain valid if the estimates are reported from unadjusted IV models with moderate confounding effects or if the treatment does not impact the outcome.

• For every simulation, we utilized a sample size of 10,000 and performed 1,000 replications.
• The true treatment effects (HR) of 3, 2, and 1 (null effect) were evaluated.
• The 95% confidence intervals (CI) were calculated as the range between the 2.5 and 97.5 percentiles of the 1000 estimates.

## Graphical abstract

**Figure:**
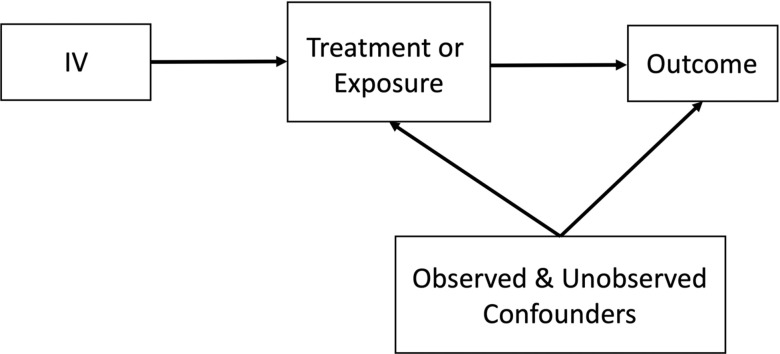

Specifications table

**Table:** 

Subject area:	Mathematics and Statistics
More specific subject area:	Causal Research
Name of your method:	Simulation studies
Name and reference of original method:	N.A.
Resource availability:	We used statistical software R version 4.0.0 for the analyses.

## Introduction

Many medical studies aim to compare treatment effectiveness, i.e., the causal relationship, and randomized controlled trials are the gold standard for doing so. However, because of ethical constraints or cost, sometimes researchers instead use observational studies for treatment effectiveness comparison. However, observational studies often face undesirable confounding bias [1], [2], [3], [4], i.e., when examining the impact of a non-randomized intervention, treatment, or exposure, unmeasured confounding represents a significant factor of bias. [5]. When the analysis disregards factors that have an impact on both the course of treatments and the outcomes, unmeasured confounding arises. Propensity score analysis or adjusting for confounding variables in a statistical model can help minimize this bias. These techniques, however, are limited to known and documented confounders, so leaving out confounders can lead to estimation bias [2]. No one can assure that unmeasured confounding will not influence observational studies.

**Fig. 1:**
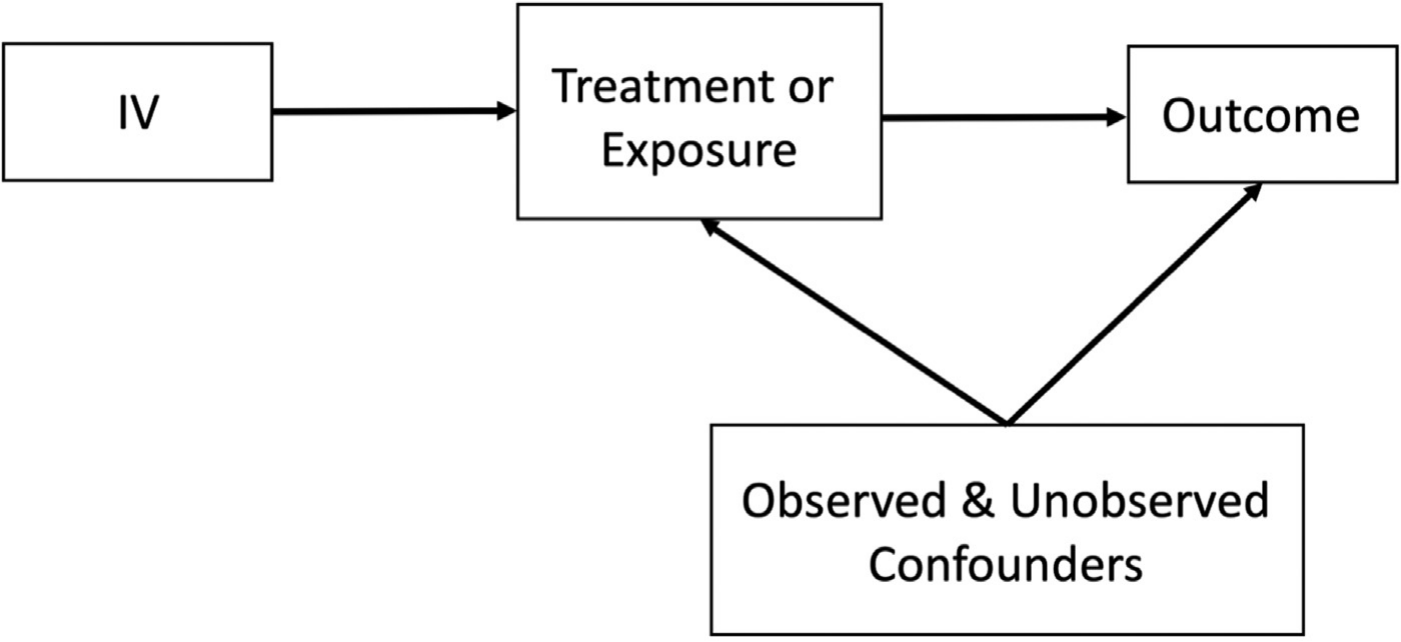
Example of the instrumental variable (IV) in a study.

The instrumental variable (IV) method, which has been applied in economics for an extended period and is now used more frequently in medical research, enables causal inferences to be drawn from observational studies even if the confounders are not measured. To do so, an IV must satisfy three fundamental assumptions, i.e., the instrumental variable should be (i) associated with the exposure of interest or treatment, (ii) not directly affect the outcome except through the exposure, and (iii) not affected by unmeasured confounders [6], [7], [8]. Fig. 1 illustrates an example of a situation where IV techniques can be used.

Implementing the IV method can be done in a variety of ways, e.g., two-stage least squares (2SLS) and two-stage residual inclusion (2SRI) [9,10]. The exposure variable is regressed on the instrument, and then the outcome variable is regressed on the predicted exposure value in 2SLS, and ordinary least squares (OLS) regression is used to fit the models at each stage [10,11]. It is a particular instance of the more general two-stage predictor substitution (2SPS) approach, which adheres to the mentioned process but may use other techniques for estimating first- and second-stage models. The 2SRI, on the other hand, includes residuals of the first stage as extra regressors in the second stage [11]. While there is some disagreement on the relative effectiveness of these two processes, 2SRI is usually regarded as having both practical and theoretical benefits [12], [13], [14].

For uncensored outcomes, IV methods are well-established. However, the IV method for survival outcomes is more complex [8,10]. For example, the 2SLS estimation method assumes that regardless of the unobserved confounders’ level, the additive effect of the exposure is equal [6,10]. This is seldom the case in survival time analysis. Moreover, the assumptions of the instrumental variables need to be satisfied, and there is censoring in survival data analysis [10]. In addition, popular survival regression models are nonlinear [8]. For survival data, probably the most widely used regression method is the Cox proportional hazards model [5,15,16]. Moreover, the Cox proportional hazard model and translating research findings into HRs are concepts that most practitioners are comfortable with [12]. For example, Tchetgen et al. introduced the IV technique for regression analysis in a survival context, particularly under an additive hazards model. The authors described two approaches—the two-stage regression approach and the control function approach—for estimating causal effects. Additionally, they demonstrate that identical techniques may be utilized under a proportional hazards model [5]. Similarly, with censored data, Li et al. showed the usefulness of the additive hazard model for IV techniques and explained the difficulties in using IV analyses with the proportional hazards model. For the additive hazard model's causal effect, they formed a closed-form, two-stage estimator [17]. For general survival data with competing risks, Ying et al. also considered the 2SRI estimator when using the additive hazards model [9]. Stukel et al. [18] found that the IV method effectively estimates treatment effects. The IV method produced an estimate more aligned with those in randomized clinical studies and was more accurate than the adjustment and propensity scores methods. However, the study did not directly model the hazard rate, and ad hoc transformations were used. This raised the issue of whether the IV technique can be developed for hazard ratios or, more generally, for calculating a Cox survival model. Later, MacKenzie et al. [2] proposed a strategy for incorporating IVs into Cox's proportional hazards model. The estimator has a significantly lower bias than the one that uses Cox's proportional hazard model and ignores omitted confounding variables. Wang et al. proposed an approach using a binary IV and a no-interaction assumption to estimate the causal hazard ratio in the presence of unmeasured confounding factors, resulting in the first consistent estimator within an instrumental variable framework [16].

However, the performance of the standard IV method for survival outcomes using the Cox model is not identified. Moreover, it is always challenging for clinicians to apply such complex methods and select the best one for their study. Therefore, the aim of this study was to assess the applicability and validity (in the context of bias and non-collapsibility of the estimates) of IV analysis using a Cox model in epidemiologic settings and compare with 2SLS and 2SRI approaches using simulated data.

## Methods

### Simulation set-up

Simulation studies were performed in several realistic scenarios with varying baseline hazards, effects of treatment on the outcome, rate of censoring, and strength of confounders. In each simulation, we considered a sample size of 10,000 with 1000 repetitions. The scenarios we considered included binary IV and continuous and binary confounders. The true treatment effects (hazard ratio, HR) of 3 (strong effect), 2 (medium effect), and 1 (null effect) were considered. We selected the notations Y for the outcome, X for exposure/treatment, Z for the IV, C for the set of observable confounding factors, and U for the set of unobserved confounding factors. The summary of the simulation settings is illustrated in Table 1.

**Table 1:** Overview of the simulation settings.

Settings	Scenarios
Sample size (n)	10,000
Number of replications in each scenario (r)	1000
Exposure (X)	Continuous
Instrumental Variable (IV)	Binary
Confounding Variables (C & U)	Continuous as well as binary
Prevalence of Treatment	20–25%
Incidence of outcome	1–10%
Prevalence of IV	40%
True treatment effect, hazard ratio (HR)	1, 2, and 3
Effect of confounders on the treatment and outcome	0.5 to 3 (small, moderate, strong)
Prevalence of Confounders	40%
Base line hazards and hazards of censoring	2–4%

### Data generation

First, we generated a continuous confounder (C1) using a multivariate normal distribution (MVND) with a mean of 0 and a variance of 1, and we dichotomized the continuous confounder using a cut-off value of 0.4 to create a binary confounder (C2). Confounders' correlation coefficients ranged from 0 to 0.2. Moreover, we simulated standard survival data (single-event data) and assumed that both potential survival time and potential censoring time followed the Weibull distribution with the scale parameter *r* = 1 (equivalent to an exponential random variable). The Weibull distribution had random censoring, and the failure rate was constant over time.

The IV was time-invariant and satisfied its basic assumptions. To obtain the average causal effect of treatment on the outcome, we also assumed that the treatment effect was homogeneous (i.e., the treatment effect is the same for all individuals in the simulated data).

### Analysis of simulated data

Treatment effects were estimated using the conventional Cox Proportional Hazard (PH) Model and two-stage IV models, in which the second-stage model was a Cox PH model [19], [20], [21]. In the two-stage model, the exposure served as the outcome variable, and the IV served as the explanatory variable in the first stage's linear regression model. The outcome was regressed on the predicted exposure rather than the actual exposure values in the second-stage model, a Cox PH model. Data were also analyzed by the two-stage residual inclusion (2SRI) approach. Parameters were estimated with and without adding confounders in the models (first stage and second stage IV), and 95% confidence intervals (CI) were estimated using 2.5 and 97.5 percentiles of the 1000 estimates. We used statistical software R for the analyses.

## Results

Table 2 compared the simulation results based on the conventional Cox PH model, Two-stage IV model, and 2SRI IV models when the strengths of confounders were moderate. Here, the effects of confounders on the exposure and outcome were 1.5 (measured) and 2.0 (unmeasured), and IV was strong enough, i.e., OR between IV and exposure was more than 4.50.

**Table 2:** Analyses based on conventional Cox model, Two-stage and 2SRI IV approach with moderate confounders.

Strength of Confounders	Prevalence of Exposure	Prevalence of Outcome	Odds Ratio between IV and Treatment	True Treatment Effect (HR)	Conventional Cox PH Model	Two-stage IV Analysis*		IV Analysis with 2SRI⁎⁎ Model	
					Adjusted HR [CI]	Unadjusted HR [CI]	Adjusted HR [CI]	Unadjusted HR [CI]	Adjusted HR [CI]
C1 =1.5 C2 = 2.0	0.24	0.09	4.55	1	1.75 [1.50–2.06]	1.00 [0.61–1.60]	0.99 [0.61–1.60]	0.66 [0.40–1.07]	0.82 [0.49–1.36]
	0.24	0.11	4.54	2	3.23 [2.80–3.73]	2.03 [1.34–2.96]	2.33 [1.50–3.62]	1.16 [0.75–1.81]	1.51 [0.94–2.44]
	0.24	0.13	4.55	3	4.55 [3.97–5.23]	2.96 [2.05–4.24]	3.73 [2.44–5.64]	1.57 [1.04–2.32]	2.14 [1.39–3.22]

2SRI: Two-stage residual inclusion; OR: Odds Ratio HR: Hazard Ratio; CI: Confidence Interval.

⁎ Standard IV model where First-stage model was a linear regression model, and the second-stage model was a Cox PH model.

⁎⁎ The two-stage residual inclusion (2SRI) includes residuals of the first stage as extra regressors in the second stage.

The unadjusted exposure effects based on the two-stage IV analyses were similar (HR= 1.00 [0.61–1.60], HR=2.03 [1.34–2.96], and HR=2.96 [2.05–4.24]) to the true value (HR=1, 2, and 3, respectively) when the strength of the confounder is moderate with the rare outcome. Nevertheless, when the IV models were adjusted for observed confounders, the effect estimates drifted away from the true value (HR=2.33 [1.50–3.62] and HR= 3.73 [2.44–5.64]) due to the non-collapsibility of the HR. For the conventional Cox PH model and 2SRI IV analysis, the effect estimates deviated (either overestimates or underestimates) from the true values. For example, when the true exposure effect was 3, the estimates from the standard Cox PH model were 4.55 [3.97–5.23]. Similarly, for the 2SRI, the adjusted estimates were 2.14 [1.39–3.22] (Table 2).

Table 3 compared the simulation results when the strengths of confounders were strong. Here, the effects of confounders on the treatment and outcome were 2.5 (measured) and 3.0 (unmeasured), and IV was strong enough, i.e., OR between IV and treatment was more than 4.00. When the strength of the confounders was strong, the effect estimates shifted away from the true values in all settings. For example, when the true treatment effect was 3, the estimate from the conventional Cox model was highly far from the true value (12.38 [10.45–14.81]), and the estimate from the standard IV model was 2.48 [1.58–3.95]. In the case of a null treatment effect, however, the estimates based on the unadjusted and adjusted IV models (only two-stage) were close to the true values (HR=1). Furthermore, a similar pattern was also found when the strength of the confounders was weak (C_1_ = 0.5; C_2_ = 1.0). For example, except for a null treatment effect, the effect estimates shifted considerably away from the true values in all settings (Supplementary Table 1).

**Table 3:** Analyses based on conventional Cox model, Two-stage and 2SRI IV approach with strong confounders (C_1_=2.5 and C_2_=3.0).

Strength of Confounders	Prevalence of Treatment	Prevalence of Outcome	Odds Ratio between IV and Treatment	True Treatment Effect (HR)	Conventional Cox Model	Two-stage IV Analysis		IV Analysis 2SRI	
					Adjusted HR [CI]	Unadjusted HR [CI]	Adjusted HR [CI]	Unadjusted HR [CI]	Adjusted HR [CI]
C_1_ = 2.5 C_2_ = 3.0	0.22	0.10	4.08	1	2.71 [2.34–3.15]	1.00 [0.63–1.59]	0.99 [0.57–1.72]	0.37 [0.21–0.60]	0.39 [0.34–1.06]
	0.22	0.12	4.09	2	4.45 [3.87–5.15]	1.84 [1.21–2.74]	2.16 [1.29–3.59]	0.58 [0.36–0.95]	0.96 [0.56–1.62]
	0.23	0.10	4.03	3	12.38 [10.45–14.81	2.36 [1.51–3.73]	2.48 [1.58–3.95]	0.95 [0.55–1.62]	0.99 [0.59–1.74]

2SRI: Two-stage residual inclusion; OR: Odds Ratio HR: Hazard Ratio; CI: Confidence Interval.

## Discussion

Healthcare databases are frequently used in clinical and health services research to compare the efficacy of drug and surgical interventions. However, these studies are vulnerable to bias because of unmeasured confounding [22], a significant potential source of bias when evaluating the effect of a nonrandomized intervention, i.e., observational studies [5]. Since two-stage IV approaches offer a straightforward strategy for assessing survival outcome studies in the presence of unmeasured confounding, they are widely used by researchers [22]. This article focused on two-stage IV and 2SRI techniques using the Cox proportional hazard model. The study's detailed simulation analysis provides useful information for researchers in selecting appropriate methodologies for empirical studies, emphasizing the significance of assessing the assumptions and limitations of IVs in Cox models when doing survival analysis.

Some studies mentioned the problem of the Cox proportional hazard model and recommended the additive hazard model as it provides a collapsible effect measure [5]. The fact that a hazard difference is a collapsible effect measure, in contrast to proportional hazards, is a key advantage of additive hazard models.

The IV technique is well established for the continuous outcome and exposure that are amenable to linear modeling. This approach has been expanded and extended to additive Aalen models [5,17] and accelerated failure time models for survival outcomes [23]. However, applying similar techniques to the more widely used Cox model has proven difficult due to the hazard ratio's non-collapsibility. This is problematic since it has been demonstrated that naive use of techniques known from basic IV models, such as 2SRI estimators under a Cox model, results in bias [24]. However, we wanted to explore the specific situations where Cox proportional hazard model may be valid in the second stage IV model.

Terza et al.'s study on the causal odds ratio indicated that the 2SRI technique is impartial when the actual model is conditional on the unmeasured confounder [25]. Earlier research [23,26] demonstrated that the bias in the treatment effect assessed using the 2SRI technique increased as the level of confounding for the treatment effect conditional on compliance or receiving treatment increased. Furthermore, analytical and simulation results from Cai et al.’s [14] study on the causal odds ratio among compliers under the principal stratification framework support such bias for the 2SRI method. The authors also demonstrated that, in contrast to the 2SRI method, the 2SPS process is biased even in the absence of unmeasured confounding.

In this study, we have found that the bias also increases in two-stage IV analyses and 2SRI approaches, and the amount of bias was highest in the 2SRI approach. Therefore, the 2SRI approach may not be a good choice for IV analysis using Cox Model.

In line with a previous study [22], we discovered that treatment effects from the two-stage IV analysis using the Cox PH model could be valid in certain circumstances, such as when confounders are moderate, and the outcome is rare. However, results from the Cox model should be interpreted with caution in empirical studies because the IV estimates are not collapsible and are prone to violations of its essential assumptions. Subject matter knowledge and evidence from data in empirical contexts should be utilized to determine the strength of the confounders in this regard.

The study's strength is its comprehensive simulation analysis, which examined the effectiveness of IV techniques for survival outcomes in the presence of unmeasured confounding. The study analysed several scenarios with varying strengths of confounders and baseline hazards and evaluated the bias and accuracy of the estimates for each scenario. The findings offer insightful information on how various IV approaches operate in various situations, which can help researchers choose the best methodologies for empirical studies. However, a limitation of our study is that we did not consider the time-varying Cox model. Moreover, due to resource constraints, we were unable to develop any applications based on actual data. In addition, the treatment effect is not homogeneous in reality as we assumed. On the other hand, the Cox proportional hazards model can be prone to non-collapsibility. In practical situations, the assumption of proportional hazards may also fail. Thus, the model we applied had such limitations. Future studies may explore these options.

## Conclusion

This study assesses the performance of the two-stage IV models, including 2SRI techniques using the Cox PH models in survival outcome studies with unmeasured confounding. The results showed that the two-stage IV approach using the Cox model might provide valid treatment effect estimates in specific situations. However, its validity depends on the strength of the IV and the degree of confounding in the data. Moreover, the estimates should be interpreted cautiously, and the IV assumptions should be carefully assessed. The 2SRI approach may not be a good choice for IV analysis using the Cox model as it may introduce more bias than the two-stage IV approach. Overall, subject matter knowledge and evidence from the data should be used to assess the strength of the confounders and choose the appropriate analytical approach.

## Ethics statement

Not applicable, as the study's outcomes were generated through simulation.

## Conference presentation statement

The abstract of the manuscript had been presented at the 31st International Conference on Pharmacoepidemiology and Therapeutic Risk Management, August 22–26, 2015, Boston, Massachusetts, USA [27].

## Funding

There was no specific grant for this study except some partial funds for the research assistant from the SUST research centre.

## CRediT authorship contribution statement

**Md. Jamal Uddin:** Conceptualization, Data curation, Formal analysis, Writing – review & editing. **Tanvir Ahammed:** Writing – original draft. **A.Z.M. Hasan Kabir:** Writing – review & editing.

## Declaration of Competing Interest

The authors declare that they have no known competing financial interests or personal relationships that could have appeared to influence the work reported in this paper.

## Data Availability

The data were generated through simulation. The “Methods” section contains a description of the simulation procedure. For this study, no extra supporting information is needed.

## Appendix Supplementary materials

Image, application 1
