# Supplementary material for: Evaluation of instrumental variable method using Cox proportional hazard model in epidemiological studies
Source: MethodsX. 2023 May 11;10:102211. doi: 10.1016/j.mex.2023.102211 (PMC10205781; doi:10.1016/j.mex.2023.102211)
Supplement: Supplementary file 1 [file mmc1.docx]

Supplementary Material

Supplementary Table 1. Analyses based on conventional Cox model, Two-stage and 2SRI IV approach with weak confounders.

| Strength of Confounders | Prevalence  of Treatment | Prevalence  of Outcome | Odds Ratio between IV and Treatment | True Treatment Effect  (HR) | Conventional  Cox Model | Two-stage IV Analysis | | IV Analysis 2SRI | |
| --- | --- | --- | --- | --- | --- | --- | --- | --- | --- |
|  |  |  |  |  | Adjusted  HR | Unadjusted  HR | Adjusted  HR | Unadjusted  HR | Adjusted  HR |
| C_1_ = 0.5  C_2_ = 1.0 | 0.20 | 0.10 | 7.70 | 1 | 1.17 | 1.00  [0.67-1.46] | 0.99  [0.66-1.48] | 0.95 | 0.97 |
|  | 0.20 | 0.12 | 7.70 | 2 | 2.30 | 2.24  [1.58-3.16] | 2.34  [1.63-3.32] | 1.79 | 1.88 |
|  | 0.20 | 0.13 | 7.67 | 3 | 3.39 | 3.67  [2.58-5.16] | 3.96  [2.77-5.63] | 2.60 | 2.80 |

2SRI: Two-stage residual inclusion; OR: Odds Ratio HR: Hazard Ratio; CI: Confidence Interval
